# Development of a liquid-phase microextraction based on the freezing of a deep eutectic solvent followed by HPLC-UV for sensitive determination of common pesticides in environmental water samples

**DOI:** 10.1039/c8ra00912k

**Published:** 2018-03-22

**Authors:** Meghdad Pirsaheb, Nazir Fattahi

**Affiliations:** Research Center for Environmental Determinants of Health (RCEDH), Kermanshah University of Medical Sciences Kermanshah Iran n.fattahi@kums.ac.ir +988338263048 +989183364311

## Abstract

In this research, a new extraction method based on liquid-phase microextraction and the freezing of deep eutectic solvent (LPME-FDES) has been developed for the determination of common pesticides in water samples prior to their analysis by high performance liquid chromatography-ultraviolet detection (HPLC-UV). In this method, a green solvent consisting of 1-octyl-3-methylimidazolium chloride and 1-undecanol was used as an extraction solvent, yielding the advantages of material stability, low density, and a suitable freezing point near room temperature. Under the optimum conditions, enrichment factors and extraction recoveries are in the range of 150–180 and 75–90%, respectively. The calibration graphs are linear in the range of 0.2–500 μg L^−1^ and limit of detections (LODs) are in the range of 0.05–0.50 μg L^−1^. Relative standard deviation (RSD) values for intra-day and inter-day of the method based on seven replicate measurements of 200 μg L^−1^ of diazinon and endosulfan, 100 μg L^−1^ of phosalone, 50.0 μg L^−1^ of atrazine, desethylatrazine and deisopropylatrazine in water were in the range of 1.3–2.5% and 2.2–3.6%, respectively. The relative recoveries of well, tap and river water samples which have been spiked with different levels of target pesticides are 97–106, 90–108 and 95–107%, respectively. The extraction methodology is simple, rapid, cheap and green since small amounts of non-toxic solvents are necessary.

## Abstract 2

In this research, a new extraction method based on liquid-phase microextraction and the freezing of deep eutectic solvent has been developed for the determination of pesticides in water prior to their analysis by HPLC-UV.

## Graphical abstract

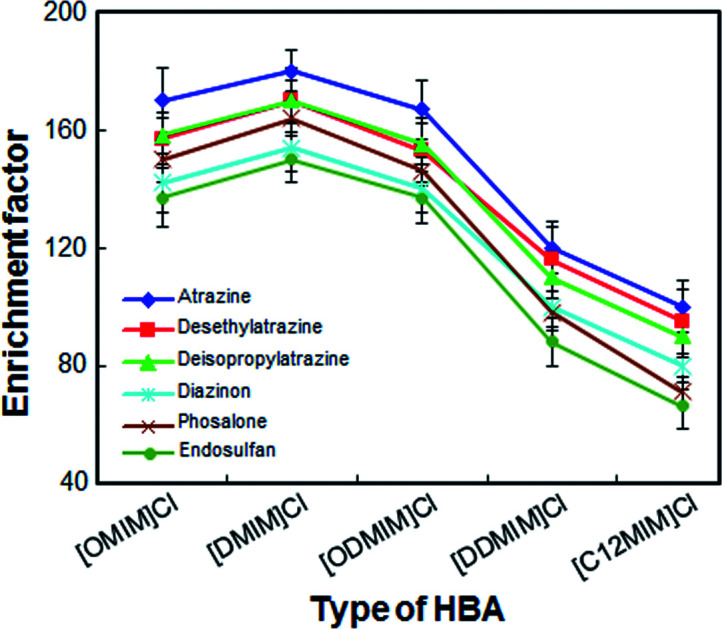

## 1 Introduction

Increasing attention has been focused on the presence of pesticides in the environment. Atrazine (2-chloro-4-ethylamino-6-isopropylamino-1,3,5-triazine; ATZ) is a herbicide belonging to the triazine family and is one of the world's most commonly used herbicides for broadleaf and grassy weed control.^1^ Atrazine is suspected as an environmental endocrine disruptor, and has caused much concern about its toxic effects on humans and ecosystems.^2^ Desethylatrazine (DEA) and deisopropylatrazine (DIA) are main metabolites of atrazine that have a longer lifetime and more toxic than atrazine.^3^ Diazinon (*O*,*O*-diethyl-*O*-2-isopropyl-6-methylpyrimidin-4-yl phosphorothioate) and phosalone (*O*,*O*-diethyl *S*-((6-chloro-2-oxobenzoxazolin-3-yl)methyl) phosphorodithioate) are two organophosphorous compounds often used in agriculture.^4^ They control a wide range of caterpillars and beetles on crops of economic importance as well as a number of hemiptera and hymenoptera.^5^ Endosulfan (6,7,8,9,10,10-hexachloro-1,5,5a,6,9,9a-hexahydro-6,9-methano-2,4,3-benzo-dioxathiepine-3-oxide) is an organochlorine pesticide. This pesticide is used for various crops in developing countries like Iran, Pakistan, India, Bangladesh, *etc.*, in order to enhance agriculture production.^6^ Nowadays, the pesticides mentioned above have been detected in various environmental matrices including soils,^7,8^ surface waters^9,10^ and groundwater.^11^ Because water quality is an important factor that affects human health, it is of great importance to develop sensitive and rapid analytical methods to monitor trace common pesticides in water.

Typically, pesticides are determined by gas chromatography,^7,12^ high-performance liquid chromatography (HPLC)^8,10,13^ and chromatography-mass spectrometry.^9,14,15^ In general, a suitable preconcentration step prior to instrument detection is necessary due to the low concentration of pesticides, and complex matrix in environmental waters. So far, many sample treatment techniques such as liquid–liquid extraction (LLE),^16^ solid phase extraction (SPE),^17^ cloud point extraction (CPE),^18^ homogeneous LLE,^19^ solid-phase microextraction,^20,21^ liquid-phase microextraction,^22,23^ dispersive liquid–liquid microextraction (DLLME)^24–26^ and dispersive liquid–liquid microextraction based on solidification of a floating organic drop (DLLME-SFO)^27–29^ have been utilized to extract pesticides from different matrices. DLLME-SFO is becoming a popular sample preparation technique, and the type of extraction solvents has been developed in recent years, *e.g.*, 1-dodecanol,^30^ 1-undecanol,^31^ hexadecane,^32^ tributyldodecylphosphonium tetrafluoroborate [P4,4,4,12][BF4],^33^ and methyltrioctylammonium hexafluorophosphate [N8,8,8,1][PF6].^34^

Recently, green and affordable extractants, called deep eutectic solvents (DESs), are being used as an alternative to common organic solvents and ionic liquids to extract low amounts of organic and inorganic compounds.^35,36^ DESs consist of two or three components with a melting point lower than their individual components, including hydrogen bond donor (HBD) such as urea, glycerol, carboxylic acid, sugar, *etc.*, and the hydrogen bond acceptor (HBA) such as quaternary ammonium salt (choline chloride) in a particular molar ratio.^37^ DESs not only have the advantages of low volatility, low vapour pressure, high thermal stability and high ability to extract organic and inorganic compounds, but also have low cost and easy preparation of non-toxic compounds. However, DESs consist of choline chloride (ChCI), urea, glycerol, carboxylic acid or sugars, which have a high hydrophilicity, which affects their use in aqueous solutions.

In this work, liquid-phase microextraction and the freezing of deep eutectic solvent (LPME-FDES) was developed to determine pesticides in environmental water samples. To our knowledge, the application of LPME-FDES for the determination of pesticides in environmental waters has not been reported until now. Low hydrophilic DES, consisting of imidazolium ionic liquid as HBA and 1-undecanol as HBD in a certain proportion, are prepared. Compared with previous DLLME-SFO,^27–29^ LPME-FDES does not require a disperser solvent.

## 2 Experimental

### 2.1 Reagents and solutions

All atrazine, diazinon, phosalone and endosulfan with a certified purity >98% were purchased from Riedel-de-Haen (Seelze, Germany). Desethylatrazine and deisopropylatrazine (97.0%) were purchased from Dr Ehrenstorfer GmbH (Augsburg, Germany). Stock standard solution of analytes was prepared in methanol (5.0 mL), with concentration of 1.00 mg mL^−1^ and was stored in a freezer at −20 °C. The working solutions were prepared daily by appropriate dilution of the stock standard solution. 1-Decyl-2,3-dimethylimidazolium chloride [DDMIM]Cl, 1-octyl-2,3-dimethylimidazolium chloride [ODMIM]Cl, 1-octyl-3-methylimidazolium chloride [OMIM]Cl, 1-dodecyl-3-methylimidazolium chloride [C12MIM]Cl and 1-decyl-3-methylimidazolium chloride [DMIM]Cl as HBA, were purchased from Sigma (St. Louis, MO, USA). The 1-undecanol as HBD, and other chemicals and reagents were supplied from Merck (Darmstadt, Germany). Ultrapure water further purified through reverse osmosis (0.055 μS cm^−1^, Millipore) was used as the working medium. Well, tap and Gharasoo River water samples, used for development of the method, were collected from Miandarband and Mahidasht (Kermanshah, Iran) in glass bottles and stored in the dark at 4 °C and analyzed within 48 h of collection.

### 2.2 Instrumentation

Chromatographic separations were carried out on an HPLC Knauer with Chromgate software version 3.1 having binary pumps Smartline-1000-1 and Smartline-1000-2, and detector Smartline-UV-2500 variable wavelength programmable (Berlin, Germany), an on-line solvent vacuum degasser and manual sample injector fitted with a 20 μL injection loop (model 7725i, Rheodyne, Cotati, CA, USA). Separations were carried out on an H5-ODS C18 column (15 cm × 4.6 mm, with 5 μm particle size) from Anachem (Luton, UK). The separation gradient employed an initial mobile phase composition of 40 : 60 (v/v) methanol : water, followed by a linear increase to 100% methanol over 20 min at a flow rate of 1 mL min^−1^ and the analytes were detected at 210 (desethylatrazine and deisopropylatrazine), 220 (atrazine) and 254 (diazinon, phosalone and endosulfan) nm.

### 2.3 Preparation of hydrophobic DESs

In this work, five imidazolium chloride ionic liquids as HBA, were mixed with 1-undecanol as HBD in different molar ratios *i.e.* 1 : 1, 1 : 2, 1 : 3, 2 : 5, 3 : 7 in a 20 mL polypropylene tube. The polypropylene tube was kept primarily sealed with a black screw cap to maintain the temperature at 40 °C with a stirring rate at 600 rpm for 20 min. After processing, a clear liquid was obtained. Among them, [DMIM]Cl was chosen as HBA because of better extraction power.

### 2.4 LPME-FDES procedure

For the LPME-FDES, an aliquot of 10.0 mL of an aqueous solution containing 200 μg L^−1^ of diazinon and endosulfan, 100 μg L^−1^ of phosalone, 50.0 μg L^−1^ of atrazine, desethylatrazine and deisopropylatrazine was placed in a 20 mL screw cap glass test tube, and 50 μL of DES as the extraction solvent was rapidly injected into the sample solution with a 100 μL syringe (gastight, Hamilton, Reno, NV, USA). The tube was then sealed and maintained at 45 °C in a water bath. After 500 mg of NaCl was added into the solution to break the emulsion, the mixture was shaken using a vortex agitator for 3 min to ensure full contact of the extractant and target compounds from the sample solution. The mixture was then centrifuged for 4 min at 5000 rpm in order to separate the mixture to phases. After centrifugation, due to difference in the density between the aqueous phase and DES, the fine droplets of DES float at the top of the test tube. The test tube was transferred into an ice bath and the DES was solidified after 5 min. Then the obtained solidified DES was transferred into a conical vial and evaporated to dryness using a stream of nitrogen at 50 °C with elimination of the aqueous phase. Finally, the DES was diluted in 30 μL of mobile phase, and 25.0 μL of the collected organic solvent containing the target compounds was injected into the HPLC-UV for analysis.

## 3 Results and discussion

### 3.1 Selection of the deep eutectic solvent

**Fig. 1:**
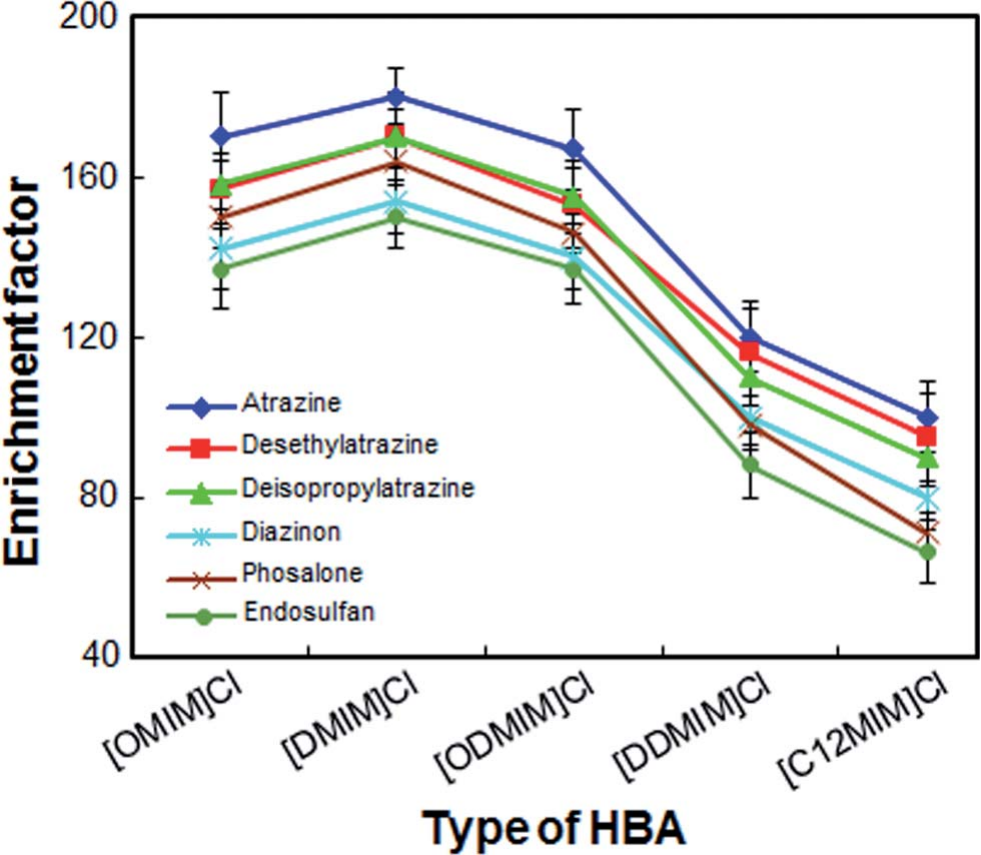
Effect of the different types of hydrogen bond acceptors on the enrichment factor of the analytes. Extraction conditions: volume of the sample solution, 10 mL; volume of the extraction solvent, 50 μL; extraction temperature, 45 °C; amount of sodium chloride, 500 mg; vortex time, 3 min; centrifugation time, 4 min; centrifuge speed, 5000 rpm.

Considering the solubility, dispersion power and extraction efficiency of DESs, selecting an appropriate extractant for LPME-FDES is especially important. Five ionic liquids of imidazolium chloride were chosen as HBAs and these ionic liquids have advantages such as low toxicity, low cost and easy to operate. In the present study, ionic liquids of imidazolium chloride were mixed with 1-undecanol in a mole ratio of 1 to 2. As shown in Fig. 1, [DMIM]Cl as HBA, shows a better enrichment factor for all analytes than other DESs. Although [OMIM]Cl and [ODMIM]Cl also have a high enrichment factor (140–170) for most analytes, the volume of these extractors, which is collected in the solidification step, is relatively small. The result may due to the partial decomposition of these two extractants caused by weak hydrogen bonding forces. The other DESs enrichment factor for the analytes is not more than 100, because the dispersion of these DESs is not very strong in the absence of a disperser solvent and they do not disperse well. As a result, [DMIM]Cl was chosen as HBA.

### 3.2 Effect of the molar ratio of HBA to HBD

**Fig. 2:**
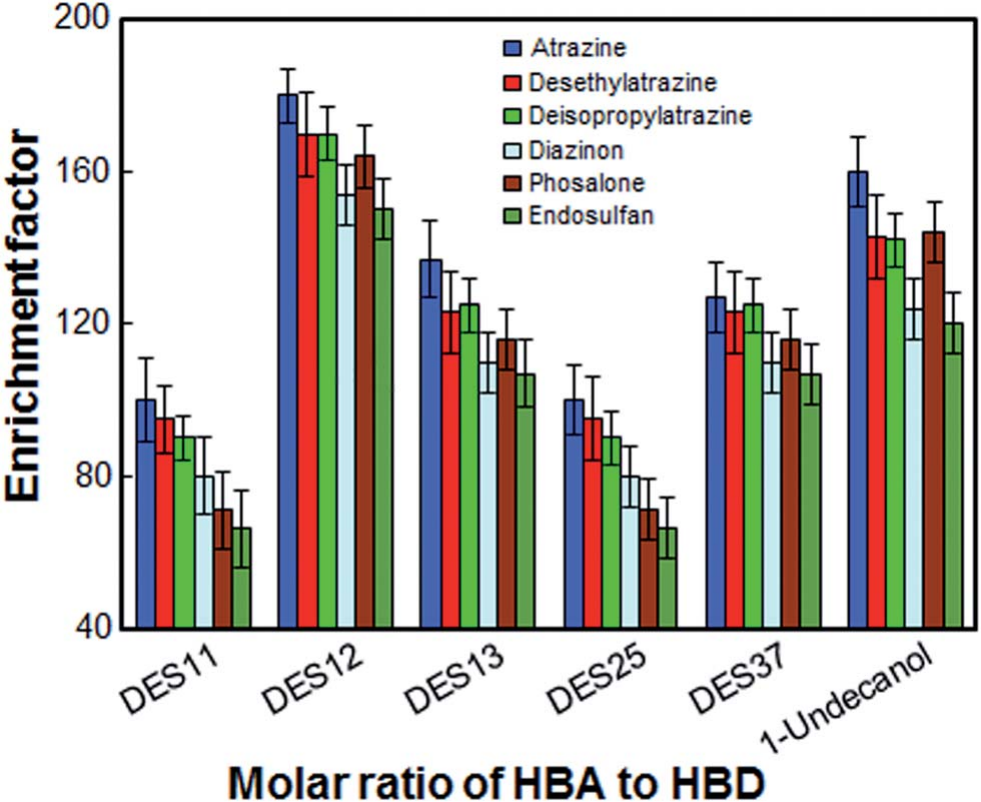
Effect of the molar ratio of hydrogen bond acceptors to hydrogen bond donor on the enrichment factor of the analytes. Extraction conditions: as in Fig. 1; type of HBA, [DMIM]Cl.

In the present work, the most suitable molar ratio for HBA to HBD was obtained to achieve high extraction efficiency. For this purpose, the extraction solvent was selected by using 1-undecanol and [DMIM]Cl with different ratios of 1 : 1 (DES11), 1 : 2 (DES12), 1 : 3 (DES13), 2 : 5 (DES25) and 3 : 7 (DES37). The results in Fig. 2 show that all extraction solvents except 1 : 1 ratio, have a positive effect on the extraction of the desired analytes. The extraction solvent with a molar ratio of 1 : 1 is gelatinous at room temperature and cannot be completely dispersed in the aqueous phase. Although the extraction efficiency of 1-undecanol is also high, DES12 exhibits better extraction efficiency and relative standard deviations compared to 1-undecanol. Thus, the optimal ratio of [DMIM]Cl to 1-undecanol was chosen to be 1 : 2.

### 3.3 Selection of the extraction solvent volume

**Fig. 3:**
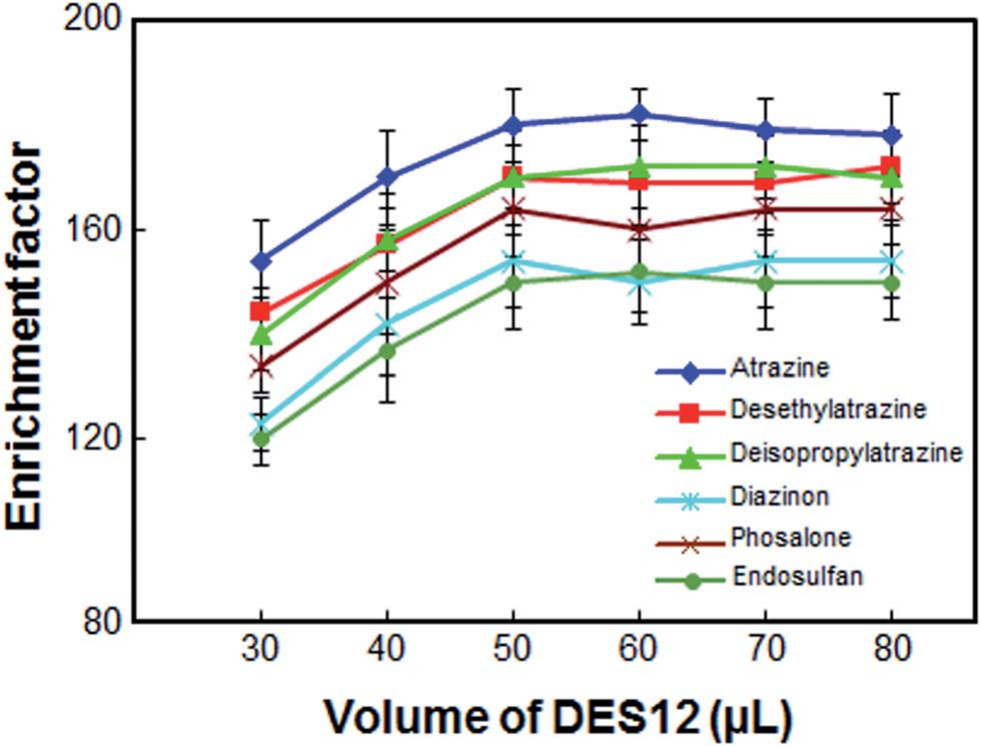
Effect of the volume of DES12 on the enrichment factor of the analytes. Extraction conditions: as in Fig. 1; type of HBA, [DMIM]Cl.

The volume of DES12 was another important parameter, as this volume directly affected the extraction efficiency. To select the optimum volume of DES12, several experiments were performed using different volumes of DES12, *i.e.* 30, 40, 50, 60, 70 and 80 μL. As can be seen from Fig. 3, the enrichment factor was enhanced by increasing the volume of DES12 from 30 to 50 μL. The enrichment factor remained almost constant when the volume of DES12 exceeded 50 μL. A volume of less than 30 μL of DES12 resulted in a final volume less than 25 μL which was insufficient for determination by the HPLC and, as a result, a systematic error would be generated. Thus, in order to have a high enrichment factor and good repeatability, 50 μL of DES12 was selected as the DES12 optimum volume.

### 3.4 Effect of the sample solution pH

**Fig. 4:**
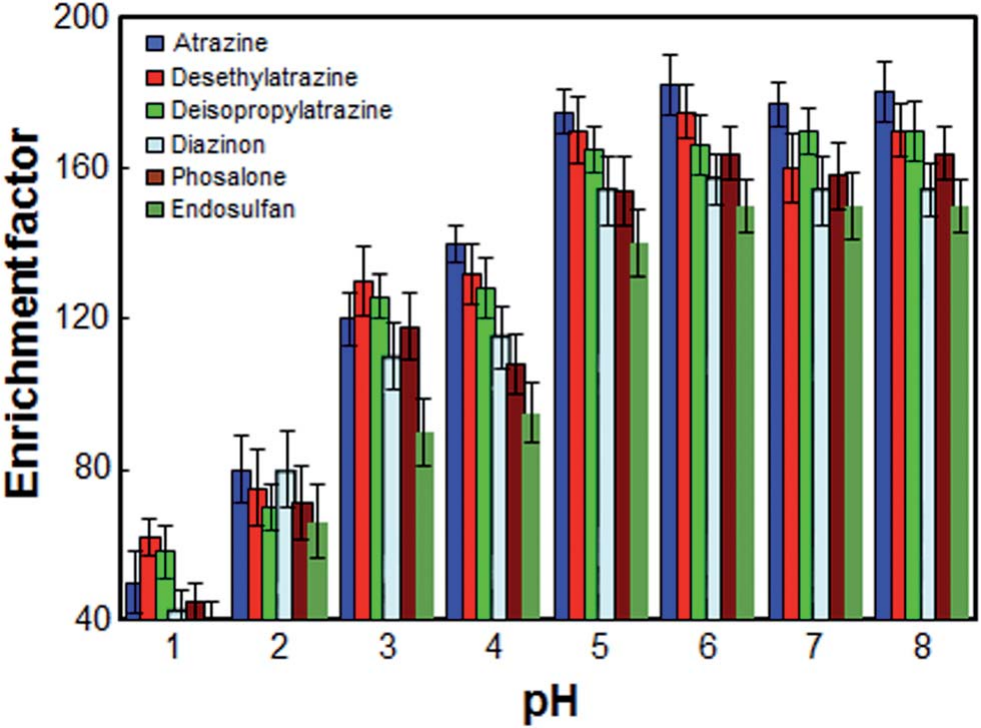
Effect of the pH on the enrichment factor of the analytes. Extraction conditions: as in Fig. 1; type of HBA, [DMIM]Cl.

The pH of sample solution is an important factor, which may affect the extraction recovery of organic compounds from water samples. The effect of pH on the pesticides extraction from water samples was studied within the range of 1–8. The results shown in Fig. 4 revealed that the enrichment factor was enhanced by increasing the pH of sample solution from 1 to 5 and remained more or less constant at higher pH values. On the other hand, since an aqueous solution of target pesticides is nearly neutral solution, within the optimized pH range (*i.e.*, pH 6.5 in 10.0 mL aqueous solution), in this work, the use of an acidic or basic solution for the pH adjustment was not needed.

### 3.5 Effect of the salt addition

The effect of salt addition on the recoveries was studied by adding different amounts of NaCl. Compared to conventional ionic liquid DLLME, during the rapid injection of DES into the aqueous phase, a water-in-row emulsion will be formed. However, this mixture creates a problem for collecting the extractant phase and leads to a reduction in the extraction efficiency of the analytes by extraction solvent. Adding the appropriate amount of sodium chloride is required to break the emulsion. When no salt was added to the solution, poor analytes recoveries were observed because the DES phase could not be completely separated from the sample solution and less extractant was collected. The amount of sodium chloride added ranged from 100 mg to 700 mg at 100 mg intervals. The results showed that when increasing the amount of salt up to 500 mg, the recovery of analytes increased and then decreased with the addition of salt. It seems that in low amounts of salt, the emulsion does not break well, and the phase separation does not completely occur. Meanwhile, high amounts of salt reduce the extraction efficiency, probably because of the decrease in the distribution coefficient of the analytes desired in the DES, due to increased ionic strength of the solution. Therefore, 500 mg of NaCl was selected as the optimum amount of salt to obtain maximum enrichment factors ranged from 150–180, based on the results.

### 3.6 Effect of the vortex time

In the proposed method, the main role of vortex time is the complete dispersion of DES12 in the aqueous sample to enhance the extraction process and the effect of durability of two unmixable phases in demulsification, simultaneously. The vortex time was studied in the range of 0 to 5 minutes. It was found that the enrichment factor of the analytes increased with increasing vortex time from 0 to 3 minutes and, with increasing vortex time, enrichment factor is constant (150–180). Before the mixture is centrifuged for the separation of the cloud system, the demulsification process is more important than the complete equilibrium of the analyte phase in an unmixable two-phase mixture. This method requires a sufficient vortex time to complete the demulsification process. Thus, 3 minutes vortex time was used in subsequent experiments.

### 3.7 Effect of the temperature

The proper temperature can accelerate the mass transfer and increase the contact surface of the extractant and the solution. In the present study, the temperature of the solution was changed in the range of 25 to 55 °C at 10 °C intervals. With increasing bath temperature to 45 °C, the enrichment factor increased and the best performance was obtained at 45 °C. At the solution temperature of more than 45 °C, the enrichment factor decreased as the solubility of the desired analytes and DES12 increased in water. As a result, 45 °C was selected as the optimum extraction temperature to obtain maximum enrichment factors ranged from 150–180.

### 3.8 Analytical figures of merit

Under the as-described optimal conditions, some major analytical performance traits of the proposed method was examined and listed in Table 1; they are as follows: linear ranges, detection limits, repeatability (intra-day), reproducibility (inter-day), enrichment factor and extraction recovery are listed. By and large, repeatability and reproducibility of a method is calculated based on seven replicate measurements. Thus, the repeatability and reproducibility of the DLLME-SDES coupled with HPLC-UV for 200 μg L^−1^ of diazinon and endosulfan, 100 μg L^−1^ of phosalone, 50.0 μg L^−1^ of atrazine, desethylatrazine and deisopropylatrazine were determined to be 1.3–2.5 and 2.2–3.6%, respectively. The calibration graph was linear in the range of 0.20–500 μg L^−1^ of target pesticides with correlation coefficient better than 0.9982. The limit of detections (LODs), based on signal-to noise ratio (S/N) of 3 ranged from 0.05 to 0.50 μg L^−1^ which is much lower than the maximum residues levels (MRLs) established by European Union regulation (ranged from 3–5 μg L^−1^ for target analytes in drinking water).^38,39^ The enrichment factors and the extraction recovery of target pesticides were from 150 to 180 and 75 to 90%, respectively.

**Table 1:** Analytical characteristics of LPME-FDES-HPLC-UV for determination of common pesticides from water sample

Compounds	RSD^a^ (intra-day, *n* = 7)	RSD% (inter-day, *n* = 7)	EF^b^	ER^c^ (%)	LR^d^ (μg L^−1^)	*r* ^2^	LOD^e^ (μg L^−1^)	LOQ^f^ (μg L^−1^)	Regression equation (*y* = *mx* + *c*)
Atrazine	1.6	2.2	180	90	0.2–100	0.9990	0.05	0.15	*y* = 182.6*x* + 2205
Desethylatrazine	2.2	2.6	170	85	0.2–100	0.9988	0.05	0.15	*y* = 203*x* + 2525
Deisopropylatrazine	1.9	3.2	170	85	0.2–100	0.9995	0.05	0.15	*y* = 193*x* + 2413
Diazinon	1.3	2.8	154	77	2–500	0.9985	0.5	1.6	*y* = 1776*x* + 21 462
Phosalone	2.4	3.1	164	82	0.5–200	0.9991	0.2	0.7	*y* = 734*x* + 5387
Endosulfan	2.5	3.6	150	75	2–500	0.9982	0.5	1.6	*y* = 1622*x* + 31 276

a Percent relative standard deviation for seven replicate measurements of the pesticides with a concentration of 200 μg L^−1^ for diazinon and endosulfan, 100 μg L^−1^ for phosalone, and 50.0 μg L^−1^ for atrazine, desethylatrazine and deisopropylatrazine.

b EF, enrichment factor.

c ER, extraction recovery.

d LR, linear range.

e LOD, limit of detection for S/N = 3.

f LOQ, limit of quantification for S/N = 10.

### 3.9 Real water analysis

**Fig. 5:**
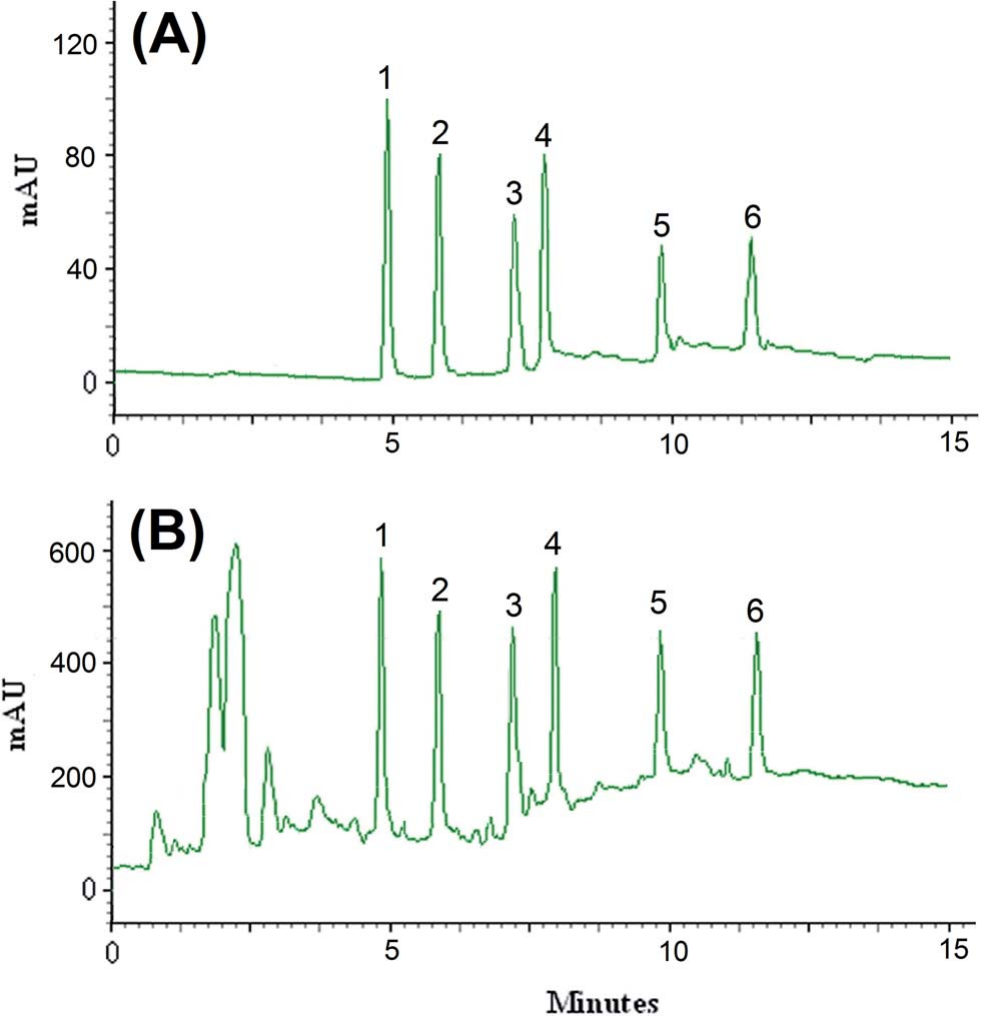
Chromatograms of direct injection of standards at concentration level of 200 μg L^−1^ of diazinon and endosulfan, 100 μg L^−1^ of phosalone, 50.0 μg L^−1^ of atrazine, desethylatrazine and deisopropylatrazine (A) and spiked river water at concentration level of 50 μg L^−1^ of diazinon and endosulfan, 25 μg L^−1^ of phosalone, 12.5 μg L^−1^ of atrazine, desethylatrazine and deisopropylatrazine (B) obtained by using LPME-FDES combined HPLC-UV. Extraction conditions: as in Fig. 1; type of HBA, [DMIM]Cl; peak identification: (1) diazinon, (2) phosalone, (3) endosulfan, (4) desethylatrazine, (5) deisopropylatrazine, (6) atrazine.

The efficiency of the LPME-FDES method was validated with the monitoring of the common pesticides in environmental water samples. River water was collected from Gharasoo River, and tap and well water samples were collected from Mahidasht and Miandarband (Kermanshah, Iran). All of water samples analyzed within 48 h of collection. The results showed that the analyzed samples were free of pesticides contamination. The samples were spiked with the standards of these pesticides at different concentration levels to assess matrix effects. Fig. 5 shows the obtained chromatograms of direct injection of standards at concentration level of 200 μg L^−1^ of diazinon and endosulfan, 100 μg L^−1^ of phosalone, 50.0 μg L^−1^ of atrazine, desethylatrazine and deisopropylatrazine (A) and spiked river water at concentration level of 50 μg L^−1^ of diazinon and endosulfan, 25 μg L^−1^ of phosalone, 12.5 μg L^−1^ of atrazine, desethylatrazine and deisopropylatrazine (B). The results of relative recovery of well, tap and river water samples are shown in Table 2. Relative recoveries for all pesticides in well, tap and river water are between 97–106, 90–108 and 95–107%, respectively. These results demonstrated that the matrices of the analyzed real water samples possess negligible effect on the proposed LPME-FDES followed by HPLC-UV determination of the pesticides.

**Table 2:** Summary of results from analysis of common pesticides in different water samples together with relative and spiking recovery

Compounds	Tap water			Well water			River water		
	Added (μg L^−1^)	Found (SD, *n* = 3) (μg L^−1^)	Relative recovery (%)	Added (μg L^−1^)	Found (SD, *n* = 3) (μg L^−1^)	Relative recovery (%)	Added (μg L^−1^)	Found (SD, *n* = 3) (μg L^−1^)	Relative recovery (%)
Atrazine	0.50	0.53 (0.06)	106	2.50	2.42 (0.12)	97	12.5	13.4 (1.3)	107
Desethylatrazine	0.50	0.45 (0.05)	90	2.50	2.58 (0.23)	103	12.5	11.96 (0.7)	96
Deisopropylatrazine	0.50	0.49 (0.08)	98	2.50	2.55 (0.20)	102	12.5	12.03 (2.4)	96
Diazinon	2.00	1.90 (0.12)	95	10.0	10.6 (0.90)	106	50	53 (3.6)	106
Phosalone	1.00	1.08 (0.15)	108	5.00	4.88 (0.35)	98	25	23.8 (2.2)	95
Endosulfan	2.00	2.10 (0.17)	105	10.0	9.96 (1.04)	100	50	50.4 (3.4)	101

### 3.10 Comparison of LPME-FDES with other methods

A comparison between the analytical performance data of this method and some microextraction techniques for the extraction and determination of pesticides from the viewpoint of the limit of detection (LOD), relative standard deviation (RSD), volume of extraction solvent and sample solution, and extraction time can be seen in Table 3. As can be seen, the limit of detection of DLLME-FDES by using only 10.0 mL of sample is better or similar to other methods, except for SPE-GC-MS,^9^ which possess lower LODs than the corresponding values obtained in this work. The RSDs values in LPME-FDES were low and the extraction time was relatively short, except for DLLME technique. The proposed method has several advantages over conventional DLLME. In contrast to conventional DLLME, there is no need for disperser solvent, so less organic solvent is used. All these results indicate that LPME-FDES is a sensitive, fast, reproducible, and simple technique that can be used for the extraction and determination of organic compounds in water samples.

**Table 3:** Comparison of the present method with other microextraction methods applied for the determination of pesticides

Method	LOD^a^ (μg L^−1^)	RSD^b^ (%)	Extraction time (min)	Extraction solvent volume (μL)	Sample volume (mL)	Reference
SPE-GC-MS	0.02–0.03	3.06–6.57	15	12 000	500	9
SMS-HPLC-DAD	0.13–1.45	2.4–6.8	—	1000	50	10
CDLLME-GC-FID	0.2–0.86	3–6	2	7215	50	13
CSDFME-GC-ECD	0.005–0.50	0.9–5.5	12.5	11	10	27
SP-UFLC-MS/MS	0.1–10 (μg kg^−1^)	<20	30	5000	2 g	40
SDLLME-HPLC-UV	1.5–6.12	2.9–5.4	3	1250	8	41
DLLME-HPLC-UV	0.05–0.1	4.5–6.2	5	1028	5	42
DLLME-SFO-HPLC-UV	0.02–0.05	4.2–5.3	<5	1030	5	43
LPME-FDES-HPLC-UV	0.05–0.50	1.3–2.5	<8	50	10	This work

a LOD, limit of detection.

b RSD, relative standard deviation.

## 4 Conclusions

In this study, the new liquid-phase microextraction method with the FDES technique was coupled to measure the pesticides in the peripheral waters. This coupling enables the LPME-FDES approach to benefit both DLLME and FDES method advantages. The new DES consists of two [DMIM]Cl and 1-undecanol parts with a molar ratio of 1 to 2. DES12 was rapidly injected into the sample solution and a cloudy emulsion system was formed, which resulted in the rapid recovery of the target compounds due to contact between the extraction solvent and the sample solution. Although DES12 has the same physical properties as 1-undecanol and other ionic liquids, but it is cheaper, less toxic, less contaminating, and its synthesis is very easy. The LPME-FDES method does not require an organic solvent as disperser in comparison with other DLLME techniques, and the organic solvent consumption is very low. Other advantages of this method are simplicity, high speed, low cost, sensitivity and not using the toxic organic solvents in the extraction process.

## Conflicts of interest

There are no conflicts to declare.

## Supplementary Material

## References

[cit1] Grennan K., Strachan G., Porter A. J., Killard A. J., Smyth M. R. (2003). Anal. Chim. Acta.

[cit2] Casa-Resino I. D., Valdehita A., Soler F., Navas J. M., Pérez-López M. (2012). Comp. Biochem. Physiol..

[cit3] Amaral B., Araujo J. A., Peralta-Zamora P. G., Nagata N. (2014). Microchem. J..

[cit4] Drufovka K., Danevcic T., Trebse P., Stopar D. (2008). Int. Biodeterior. Biodegrad..

[cit5] Alizadeh A., Talebi K., Hosseininaveh V., Ghadamyari M. (2011). Pestic. Biochem. Physiol..

[cit6] Memon S., Memon N., Memon S., Latif Y. (2011). J. Hazard. Mater..

[cit7] Yu Y., Liu X., He Z., Wang L., Luo M., Peng Y., Zhou Q. (2016). Anal. Methods.

[cit8] Kaczyński P., Łozowicka B., Jankowska M., Hrynko I. (2016). Talanta.

[cit9] Tavakoli M., Hajimahmoodi M., Shemirani F. (2014). Anal. Methods.

[cit10] Scheel G. L., Tarley C. R. T. (2017). Microchem. J..

[cit11] Korosa A., Auersperger P., Mali N. (2016). Sci. Total Environ..

[cit12] Cinelli G., Avino P., Notardonato I., Russo M. V. (2014). Anal. Methods.

[cit13] Farajzadeh M. A., Mohebbi A., Feriduni B. (2016). Anal. Chim. Acta.

[cit14] Ranz A., Eberl A., Maier E., Lankmayr E. (2008). J. Chromatogr. A.

[cit15] Zhou T., Hou J., Yuan D., Li H., Zhang P., Li Y., Ding H., Chen Y., Ding L. (2016). RSC Adv..

[cit16] Zaater M., Tahboub Y., Qasrawy S. (2005). Anal. Lett..

[cit17] Fenoll J., Hellín P., Sabater P., Flores P., Navarro S. (2012). Talanta.

[cit18] Xie S., Paau M. C., Li C. F., Xiao D., Choi M. M. F. (2010). J. Chromatogr. A.

[cit19] Takagai Y., Igarashi S. (2001). Analyst.

[cit20] Zhang G., Zang X., Li Z., Chang Q., Wang C., Wang Z. (2014). Anal. Methods.

[cit21] Saraji M., Shahvar A. (2017). Anal. Chim. Acta.

[cit22] Sobhi H. R., Ghambarian M., Behbahani M., Esrafili A. (2017). J. Chromatogr. A.

[cit23] Wu Q., Zhao Y., Wang C., Sun M., Ma X., Wang Z. (2015). Anal. Methods.

[cit24] Rezaee M., Khalilian F., Mashayekhi H. A., Fattahi N. (2014). Anal. Methods.

[cit25] Rezaee M., Mashayekhi H. A., Saleh A., Abdollahzadeh Y., Naeeni M. H., Fattahi N. (2013). J. Sep. Sci..

[cit26] Sharafi K., Fattahi N., Pirsaheb M., Yarmohamadi H., Fazlzadeh Davil M. (2015). Int. J. Cosmet. Sci..

[cit27] Karimaei M., Sharafi K., Moradi M., Ghaffari H. R., Biglari H., Arfaeinia H., Fattahi N. (2017). Anal. Methods.

[cit28] Sadeghi M., Nematifar Z., Fattahi N., Pirsaheb M., Shamsipur M. (2016). Food Anal. Method..

[cit29] Guiñez M., Martinez L. D., Fernandez L., Cerutti S. (2017). Microchem. J..

[cit30] You X., Wang S., Liu F., Shi K. (2013). J. Chromatogr. A.

[cit31] Pirsaheb M., Fattahi N., Pourhaghighat S., Shamsipur M., Sharafi K. (2015). LWT--Food Sci. Technol..

[cit32] Leong M.-I., Huang S.-D. (2009). J. Chromatogr. A.

[cit33] Hu L., Shan W., Zhang Y., Li S., Gao H., Lu R., Zhang S., Zhou W. (2016). RSC Adv..

[cit34] Wang H., Hu L., Li W., Yang X., Lu R., Zhang S., Zhou W., Gao H., Li J. (2017). Talanta.

[cit35] Paiva A., Craveiro R., Aroso I., Martins M., Reis R. L., Duarte A. R. C. (2014). ACS Sustainable Chem. Eng..

[cit36] Abbott A. P., Boothby D., Capper G., Davies D. L., Rasheed R. K. (2004). J. Am. Chem. Soc..

[cit37] Bezold F., Weinberger M. E., Minceva M. (2017). J. Chromatogr. A.

[cit38] Nagaraju D., Huang S. D. (2007). J. Chromatogr. A.

[cit39] Berijani S., Assadi Y., Anbia M., Hosseini M. R. M., Aghaee E. (2006). J. Chromatogr. A.

[cit40] Liu A., Dou X., Zhang L., Li Q., Qin J., Duan Y., Yang M. (2018). Chemosphere.

[cit41] Li S., Gao P., Zhang J., Li Y., Peng B., Gao H., Zhou W. (2012). J. Sep. Sci..

[cit42] Shamsipur M., Fattahi N., Pirsaheb M., Sharafi K. (2012). J. Sep. Sci..

[cit43] Pirsaheb M., Fattahi N., Shamsipur M., Khodadadi T. (2013). J. Sep. Sci..

